# The Relationship Between Gut Microbiota and Recurrent Spontaneous Abortion

**DOI:** 10.3390/microorganisms13051073

**Published:** 2025-05-04

**Authors:** Yiyao Huang, Ruijie Fang, Ting Xiong, Wei Li, Nan Yu

**Affiliations:** 1 Tongji Medical College, Huazhong University of Science and Technology, Wuhan 430000, China; u202110386@hust.edu.cn (Y.H.); m202476722@hust.edu.cn (R.F.); 2 Department of Obstetrics and Gynecology, Tongji Hospital, Tongji Medical College, Huazhong University of Science and Technology, Wuhan 430000, China; txiong@tjh.tjmu.edu.cn (T.X.); topliwei@126.com (W.L.)

**Keywords:** recurrent spontaneous abortion (RSA), recurrent miscarriages, gut microbiota, immunology

## Abstract

Recently, the gut microbiota has been found to be associated with multiple organs and systems in the human body, playing a key role in the occurrence and development of various diseases, such as the gut–brain axis and the gut–liver axis. However, its interaction with miscarriages remains poorly understood. This article reviews the characteristics of gut microbiota and its metabolites in patients with recurrent spontaneous abortion (RSA), the mechanism of gut microbiota inducing RSA, and potential therapeutic strategies. Therefore, it provides a new perspective on the gut microbiota in the pathogenesis and treatment of recurrent abortion, and the prospect of the future research direction of gut microbiota and recurrent abortion is proposed based on existing studies.

## 1. Gut Microbiota Composition and Function

The gut microbiota is composed of approximately 2000 species of microorganisms, with a population reaching up to 10^14^, outnumbering human body cells by a factor of ten. It contains 150 times more genomic information than the human genome [1]. These microorganisms produce millions of metabolites, actively participate in human metabolism, and maintain physiological functions. Notably, at least 20% of the small molecules in human blood are microbiota-derived products, establishing a symbiotic relationship with the host. Due to its extensive physiological influence, the gut microbiota is often regarded as a virtual organ essential to human health. With the rapid advancement of microbial molecular technology, the roles of gut microbiota in maintaining human health are increasingly being discovered, making it a research hotspot in endocrinology and immunology.

Gut microbiota can be broadly categorized into three groups: commensal microbiome, conditionally pathogenic microbiome, and pathogenic microbiome. Normal gut microbiomes are involved in nutritional metabolism, immune regulation, antimicrobial protection, intestinal mucosal growth, and regulation of physiological and psychological states [2]. Currently, the known beneficial effects of gut microbiota include aiding in the digestion of complex food components, providing beneficial metabolites such as short-chain fatty acids (SCFAs) and B vitamins [3], protecting the intestinal mucus layer and barrier, and regulating immune activity. However, factors such as diet, medication, age, and health status can affect the composition of intestinal microbiota, leading to an imbalance that affects intestinal mucosal barrier function and may cause systemic “metabolic endotoxemia” and immune and metabolic abnormalities in the host [4]. In response to the intestinal microbiome imbalance, probiotics or prebiotics can be applied to improve the symptoms of immune and metabolic abnormalities.

The adult intestinal microbiota is predominantly composed of six main groups of bacteria: Firmicutes, Bacteroidetes, Actinobacteria, Proteobacteria, Verrucomicrobia, and Fusobacteria. Of these, Firmicutes and Bacteroidetes can constitute up to 90% of the gut microbiota. Within these phyla, specific genera and species are prevalent; for example, 95% of Firmicutes are Clostridium species, while the remainder includes Lactobacillus, Bacillus, and Ruminococcus [5]. During normal pregnancy, the composition of a woman’s gut microbiota undergoes significant changes. In early pregnancy, the gut microbiota of pregnant women shows little difference from that of healthy non-pregnant women. However, in late pregnancy, the gut microbiome composition more closely resembles that of patients with metabolic syndrome [6]. There is an observed decrease in alpha diversity (within-sample diversity), an increase in beta diversity (between-sample diversity), and a decrease in certain beneficial bacteria, such as Enterobacter baumannii and Pseudomonas putida [7]. These changes are closely linked to the dramatic hormonal and metabolic fluctuations that occur during pregnancy. Altered gut microbiota and their metabolic activities can influence pregnancy outcomes by regulating the host’s metabolism and immune system. For example, Chen Y. et al. found that *Bacteroidetes*, *Proteobacteria*, *Actinobacteria*, *Escherichia*, *Streptococcus_Salivarius*, and *Lactobacillus* were significantly reduced in missed abortion (MA) patients, while *Ruminococcaceae* and *[Eubacterium]_coprostanoligenes_group* were only found in MA patients, but not in normal pregnant women [8]. This highlights the potential for early pregnancy gut microbiota alterations to disrupt maternal immune and metabolic balance, thereby increasing the risk of adverse pregnancy outcomes. Understanding these changes and their impacts is crucial for addressing complications such as recurrent spontaneous abortion.

The gut microbiota primarily decomposes various types of ingested food, converting them into metabolites through glucose and protein metabolism pathways. The microbiota and their metabolites play a crucial role in regulating nutrient absorption, fat metabolism, intestinal epithelial cell growth, and immune responses. Additionally, metabolites from gut microbiota can signal distant organs, linking gut health with the host’s immune, hormonal, neurological, and metabolic functions [9]. Studies have suggested that metabolites such as SCFAs, trimethylamine N-oxide (TMAO), bile acids, and B vitamins can significantly affect the host, particularly influencing pregnancy [10].

Gut microbiota can impact all stages of a woman’s fertility, including follicular development, embryo implantation, and the entire pregnancy process [11]. Pregnancy is a complex physiological process involving significant hormonal, immunological, and metabolic changes in the woman’s body, which in turn affect the composition of the gut microbiota. Research has shown correlations between specific types of gut microbiota during pregnancy and complications, adverse pregnancy outcomes, and even long-term neonatal health [12,13,14]. Alterations in gut microbiota may play differential roles in early and late pregnancy loss. In early pregnancy, immune tolerance toward the embryo is crucial, and gut microbiota is known to regulate immune balance via modulation of Treg/Th17 ratios and production of anti-inflammatory metabolites such as SCFAs. Dysbiosis during this phase may disrupt maternal–fetal immune tolerance and contribute to early recurrent pregnancy loss. In contrast, in later stages of pregnancy, excessive changes in gut microbiota composition resembling metabolic syndrome-like profiles may trigger systemic inflammation, increased gut permeability, and endotoxemia, thereby impairing placental function and leading to late spontaneous abortion or preterm birth. Understanding these relationships is essential for improving maternal and fetal health outcomes.

Moreover, certain clinical entities characterized by subfertility and recurrent spontaneous abortion, such as polycystic ovary syndrome (PCOS) and endometriosis, have been increasingly associated with distinct alterations in the microbiota profile. Celiac disease has also been associated with URSA, potentially due to alterations in intestinal permeability that lead to immune dysregulation and systemic inflammation [15,16]. Autoimmune hepatitis is also characterized by alterations in gut microbiota, and interventions aimed at restoring a healthy microbial profile may serve as a potential adjunctive treatment strategy [17].

## 2. Definition of Recurrent Spontaneous Abortion and Possible Mechanisms

RSA refers to the loss of two or more consecutive pregnancies with the same partner. Its etiology is complex, involving environmental influences, genetic predispositions, and various other factors. RSA is a common pregnancy complication, affecting 1% to 5% of women of reproductive age, with the risk of recurrence increasing with each successive miscarriage. Studies indicate that the rate of spontaneous abortion in RSA patients can reach 70–80% after a second pregnancy [18]. Diagnostic criteria for RSA vary internationally, differing in the required number of miscarriages, gestational age at which they occur, and confirmation of pregnancy. In China, RSA is clinically defined as two or more consecutive pregnancy losses with the same partner before 28 weeks of gestation [19]. Traditional Chinese medicine (TCM) refers to RSA as “slip of the fetus” and treats it under the concept of repeated pregnancy loss. Historically, the term “slip of the fetus” in TCM has evolved to encompass what is now recognized as RSA. Some TCM practitioners also use the term “repeated abortions” to describe this condition. Understanding these definitions and their cultural contexts is crucial for addressing RSA in diverse clinical settings.

Currently, the exact cause of more than 40% of RSA cases remains undetermined, referred to as “unexplained RSA” (URSA). It is anticipated that ongoing research will identify more independent causative factors. Presently, the known etiology of RSA is categorized into several groups: local factors of the reproductive system, comorbid endocrine disorders, thrombophilic factors, and immunologic factors. Local factors of the reproductive system include anatomical abnormalities and inflammatory diseases. Endocrine disorders contributing to RSA encompass thyroid dysfunction and abnormal glucose metabolism. Thrombophilic factors, whether hereditary with relevant gene mutations or acquired secondary to other conditions, can affect blood perfusion in the utero-placental circulation, thereby increasing RSA risk. Increasing evidence supports the role of immune dysfunction in the development of RSA, although the precise mechanisms remain unclear, and no specific diagnostic methods are available. To date, imbalances in Treg/Th17 cell ratios, M1/M2 macrophage polarization, disturbances in NK cell activity, and altered cytokine profiles have been reported in URSA [20]. Based on these findings, immune modulation has been explored as a potential therapeutic strategy for its treatment, such as immune modulation therapy, immunoglobulin therapy, anticoagulant therapy, immune adaptation therapy etc. [20]. Genetic factors and lifestyle habits also impact RSA risk. As research progresses, changes in gut microbiota and its metabolites during pregnancy have attracted widespread attention, and such changes may be related to the occurrence of RSA to a certain extent.

## 3. Differences in Gut Microbiota and Metabolites Between Recurrent Miscarriages and Normal Pregnancies

The rapid development of DNA sequencing technology has transformed our understanding of microbial communities in various complex habitats. To compare the gut microbiome of normal pregnant women with that of patients experiencing RSA, stool and other samples from both RSA patients (observation group) and healthy pregnant women (control group) were collected. These samples were cultured, and qualitative and quantitative analyses were conducted to identify differences between the groups. Additionally, 16S rDNA sequencing was employed. 16S rDNA sequencing is a high-throughput technology used to analyze bacterial community composition within specific environments or habitats. It enables the study of microbial diversity, abundance, and community structure in environmental samples, providing insights into the relationship between microorganisms and their host or environment. This article focuses on the differences in gut microbiome between RSA patients and healthy individuals, aiming to shed light on the potential impact of gut microbiota on pregnancy outcomes.

At present, there is little literature analyzing the differences in microbiome between RSA patients and normal pregnant women (Table 1). By comparing the intestinal microbiota of 63 cases in the RSA group, 60 cases in the abortion control group, and 53 cases in the pelvic surgery and non-EM group, Xia Meiyan et al. found that yeast, *Enterococci*, and *Enterobacteria* were positively correlated with RSA, while *Lactobacillus* and *Bifidobacterium* were negatively correlated with RSA [18]. Liu Y.J. et al. studied the intestinal microorganisms, fecal metabolites, and serology of patients with unexplained abortion. They found that *Prevotella spp_1*, *Prevotella spp_UCG_003*, and *Lunar Aeromonas spp_1*, which are predominant bacteria in healthy populations, were significantly reduced in the abortion group. Furthermore, the bacterial biodiversity in the abortion group was significantly lower compared to the healthy control group [21]. However, Cui Y. et al. found that the bacterial abundance index decreased in RSA patients, but the bacterial diversity index increased. They also found that *Roseburia* significantly decreased while *Ruminococcus* significantly increased in RSA patients. Furthermore, in RSA patients with intrauterine adhesion, PCOS, and BMI > 23.9, *Klebsiella* significantly increased, while *Prevotella.9* and *Roseburia* significantly decreased [22].

A study selected the top 30 most abundant microbial species for categorization and found significant differences in microbial composition and abundance between the observation group (RSA patients) and the control group (healthy individuals) at both the phylum and species levels. In the observation group, the top five dominant genera at the species level were:

*Clostridium_sensu_stricto_1_unclassified*

*Escherichia-Shigella_unclassified*

*Klebsiella_pneumoniae*

*Streptococcus_salivarius*

*Uncultured_Klebsiella_sp.*

In contrast, the top five dominant genera in the control group were:

*Megamonas_unclassified*

*Bacteroides_unclassified*

*Agathobacter_unclassified*

*Faecalibacterium_unclassified*

*Bacteroides_uniformis* [3]

More clinical trials’ results are shown in Table 1. In addition, a Mendelian randomization (MR) analysis showed that *Coprococcus3* and *Odoribacter* were linked to a reduced risk of RSA, while the *Eubacterium ruminantium* group was associated with an increased risk of RSA [23]. These findings highlight the distinct differences in gut microbial composition between RSA patients and healthy individuals, suggesting that specific microbial profiles may be associated with recurrent spontaneous abortion. Understanding these differences could provide insights into potential diagnostic markers and therapeutic targets for RSA.

In addition to gut microorganisms, other dysbiosis—defined as an imbalance in the microbial community structure—such as vaginal and endometrial microbiota can also impact RSA. Twenty-nine microorganisms have been isolated from female vaginal secretions, with *Lactobacillus* being the most abundant and important gram-positive bacillus. These include species like Lactobacillus iners, *Lactobacillus gasseri*, *Lactobacillus crispatus*, and *Lactobacillus jensenii* [24]. It has been reported that a *Lactobacillus curvatus*-dominated vaginal microenvironment is more stable and less prone to bacterial vaginosis, which can contribute to healthier pregnancy outcomes [25].

The endometrial microbiota also plays a role in RSA. The presence of Proteobacteria in the endometrial microbiota has been positively correlated with RSA, whereas the presence of *Sphaerita-Thermophilus* is negatively correlated [18]. However, it has been suggested that the microbiota composition in endometrial fluid may not fully reflect the microbial organization within the endometrium. Comprehensive sampling from both endometrial fluid and biopsies is necessary to gain a complete understanding of microbial colonization and its relationship with RSA [26].

## 4. Possible Mechanisms of Influence of Intestinal Microbiota on Recurrent Miscarriages

In recent years, evidence has increasingly supported the role of the gut-endometrial axis in the pathogenesis of early pregnancy complications. The gut microbiota, influenced by factors such as host genetics, immune system, and diet, undergoes significant changes during pregnancy. These changes are linked to the host’s physiological and immune adaptations, with bacteria potentially translocating from the gut to placental tissues via dendritic cells. The remodeling of the microbiota during pregnancy is a positive maternal response to promote necessary immune and metabolic adaptations for a successful pregnancy. However, deviations in gut microbiota composition can predispose mothers to physiological maladaptation, influencing pregnancy outcomes either beneficially or detrimentally, potentially leading to miscarriage [1]. Altered maternal gut microbes can trigger immune responses through mechanisms such as adhesion to the gut epithelial barrier, translocation, or the release of mediators like lipopolysaccharides (LPS), SCFAs, or extracellular vesicles (EV). At the maternal–fetal interface, such disturbances can lead to a tolerogenic environment shift, characterized by an increased pro-inflammatory macrophage (M1) phenotype and elevated levels of pro-inflammatory cytokines, including tumor necrosis factor-α (TNF-α), interleukin (IL)-6, and IL-1β. Concurrently, reductions in regulatory T cells (Tregs), regulatory B cells (Bregs), follicular regulatory T cells (Tfr), transforming growth factor-β (TGF-β), and IL-10 have been observed during pregnancy, contributing to various pregnancy complications.

This growing body of research suggests that remodeling the host gut microbiota and altering its metabolism can significantly influence pregnancy outcomes. Understanding these mechanisms further may provide insights for the development of effective interventions to improve maternal and fetal health.

### 4.1. Immune Response

Among the few recognized causes of recurrent miscarriage, immune factors stand out as the most significant. Over the past few decades, the connection between autoantibodies and pregnancy loss has garnered widespread attention. The primary functions of gut microbiota include strengthening the epithelial barrier, enhancing mucosal adhesion while inhibiting pathogen adhesion, competitively eliminating pathogens, producing antimicrobial substances, modulating dendritic cells (DCs), affecting T-cell polarity, and regulating inflammation [27].

#### 4.1.1. Inflammatory Response

**Figure 1:**
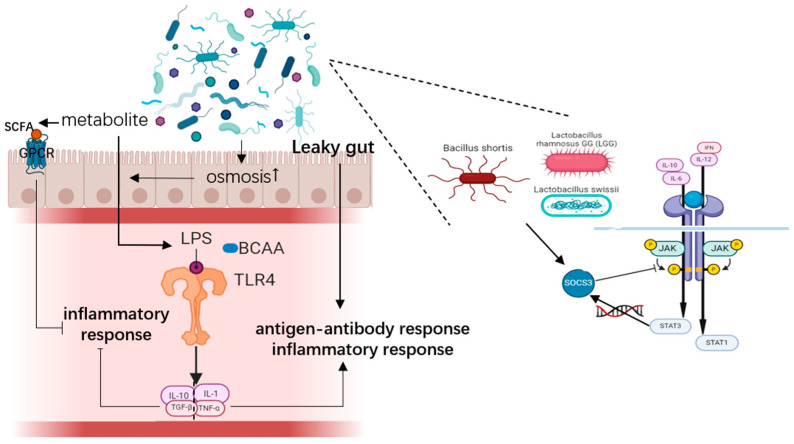
Gut microbiota and metabolite trigging inflammatory response (Created in BioRender. Yiyao Huang (2024) https://BioRender.com).

First, the upregulation of several pro-inflammatory cytokines in the peripheral blood and meconium of RSA patients is a key mechanism contributing to RSA, closely linked to chronic inflammation at the maternal or maternal–fetal interface (Figure 1). Normal levels of cytokines and chemokines are crucial for a favorable pregnancy outcome [28]. For instance, IL-10 helps to inhibit inflammatory cytokines and protects the fetal–placental unit [29]. It works alongside IL-4 and IL-13 to regulate trophoblastic invasion [30] and may stimulate placental angiogenesis by inducing trophoblasts to produce vascular endothelial growth factor-C (VEGF-C) and aquaporin (AQP1) systems [31]. Conversely, the inflammatory cytokine TNF-α increases during pregnancy, primarily originating from the placenta. Elevated TNF-α levels can worsen insulin resistance, which is associated with the development of gestational diabetes mellitus (GDM).

The levels of IFNthese cytokines can be regulated by the cytokine signaling protein (SOCS) family. Studies have shown that *Bacillus shortis*, *Lactobacillus rhamnosus GG* (*LGG*), and *Lactobacillus swissii* induce SOCS3 macrophage expression [32]. SOCS proteins act as negative regulators of cytokine signaling pathways by activating and phosphorylating SMART STAT-dependent JAK transcription factors. Typically, STAT1 is associated with signaling interferon (IFN) and IL-12, while STAT3 is linked to anti-inflammatory signals IL-10 and IL-6. SOCS3, induced by IL-10 and IL-6, can inhibit the expression of pro-inflammatory factor genes and the negative signaling of IL-10 and IL-6 [27].

Various inflammatory mediators produced by gut microbiota, such as LPS and branched-chain amino acids (BCAA), can trigger an immune response. These mediators activate Toll-like receptor (TLR) 4, initiating a range of cellular signaling pathways that stimulate the production of inflammatory factors, including IL-1, IL-10, and TNF-α [1]. Dysbiosis, an imbalance in the gut microbiota, increases intestinal mucosa permeability, allowing more LPS to enter the circulation—a phenomenon known as leaky gut. This leads to an antigen–antibody response and activation of the inflammatory response system [33].

SCFAs including acetate, propionate, and butyrate produced from carbohydrate fermentation (e.g., dietary fiber) and by microbial metabolites are nontoxic to the host and serve as an important energy source for epithelial cells. These metabolites are primarily transported through the colon and distal colon, as they are not readily absorbed or digested. SCFAs and other bacterial metabolites activate G protein-coupled receptors (GPCRs) on intestinal epithelial cells (IECs), which helps reduce the host’s inflammatory response. Additionally, intact metabolites can stimulate Toll-like receptors on IECs and host immune cells, inducing the production of anti-inflammatory cytokines such as IL-10 and TGF-β. Another laboratory study has demonstrated that exposure to these substances can lead to the release of cytokines and chemokines in intestinal epithelial cells, monocytes, and DCs [34,35].

It has also been suggested that psychological stress can also disrupt the balance between gut microbiota and host mucosal integrity, and that gut microbiota metabolites contribute to miscarriage by enhancing stress-triggered immune activation. Psychosocial stress enhances gastrointestinal permeability and absorptive gut bacteria. LPS in the gastrointestinal tract becomes endogenous LPS through TLR4 as a danger signal [36].

#### 4.1.2. T Cell Homeostasis

Dysregulation of Th1/Th2 and Th17/Treg balance is a major factor in RSA. Numerous studies have demonstrated a close relationship between the intestinal microbiome and the balance of Th17 and Treg cells [27]. Helper T cell (Th) 1 differentiates from Th0 cells in response to IL-12 and other cytokines. These CD4^+^ cells secrete IL-2, IFN-γ, and TNF-β, and are primarily involved in immune responses against intracellular bacteria and protozoa. Th2 is another CD4^+^ T cell subset that secretes Th2-type cytokines, such as IL-4, IL-5, IL-10, and IL-13. Th2 cells assist in B cell activation and play a key role in humoral immunity. Th17 is a newly identified T cell subset that produces IL-17. Differentiated from Th0 cells under the influence of IL-6 and IL-23, Th17 cells mediate neutrophil mobilization and contribute to tissue inflammation. Th17 cells also play a significant role in autoimmune diseases [27]. Tregs, which are CD4^+^ and Foxp3^+^, are crucial for regulating immune responses. They function by directly inhibiting target cell activation and by secreting cytokines such as TGF-β and IL-10, which suppress the immune response.

**Figure 2:**
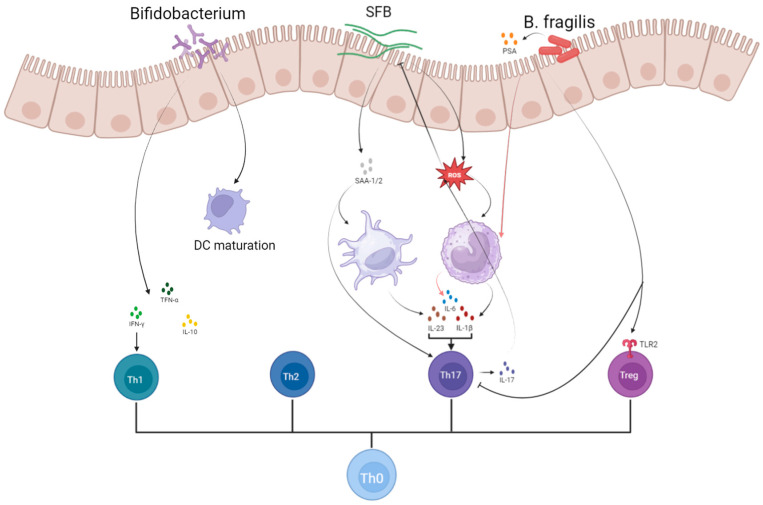
Several gut microbiota influencing T cell differentiation (Created in BioRender. Yiyao Huang. (2024)).

**Figure 3:**
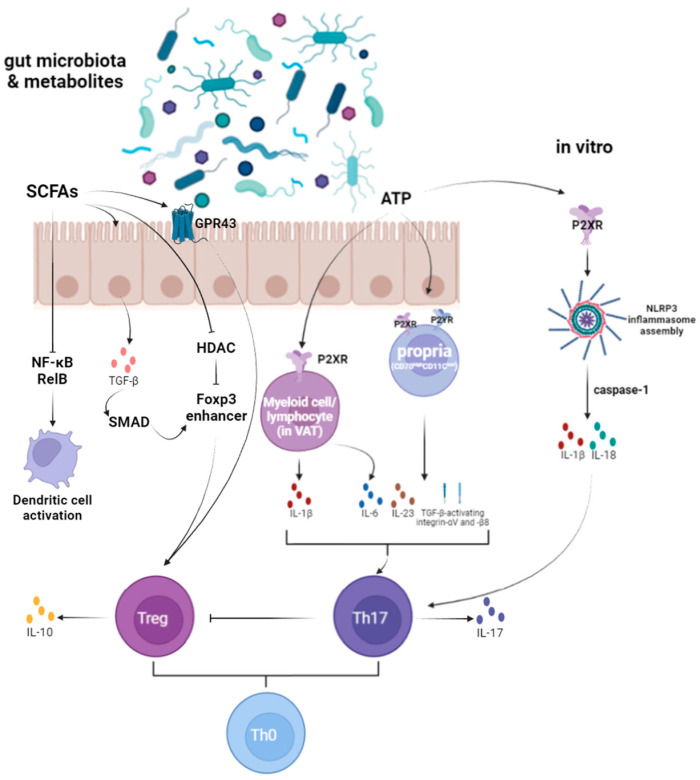
Gut microbiota metabolites affecting T cell differentiation (Created in BioRender. Yiyao Huang. (2024)).

Normal pregnancy represents a unique form of maternal immune tolerance, characterized by a Th2-dominated immune response. If this balance shifts towards a Th1 response, it can lead to over-activation of CD8^+^ T cells and natural killer (NK) cells, increasing the risk of miscarriage. Disruption of the dominant intestinal microbiota can favor a Th1-mediated immune response. (Figure 2) Studies have demonstrated a positive linear correlation between gut microbiota dysbiosis and Th1/Th2 balance, as well as a positive correlation between microbial metabolites and changes in Th1/Th17 cytokine levels (Figure 3) [1].

*Segmented filamentous bacteria* (*SFB*) play a crucial role in inducing and maintaining Th17 cells in the lamina propria (LP), which are essential for mucosal protection [37,38,39]. Research by Ivanov et al. found that the differentiation of Th17 cells correlates with the presence of *SFB* in both Th17 cell-sufficient and Th17 cell-deficient mice [40]. Further studies revealed that *SFB* attachment to ileal epithelial cells stimulates the production of reactive oxygen species (ROS), which enhances IL-1β secretion and promotes Th17 cell differentiation [41,42]. *SFB* colonization also leads to the production of serum amyloid A1 and A2 (SAA), which acts on LP dendritic cells to increase local IL-17A expression in RORγt^+^ T cells and facilitate Th17 cell differentiation in vitro [42,43]. Another study demonstrated that CD11C^+^ MHCII^+^ monocyte-derived cells, a subset of antigen-presenting cells, secrete significant amounts of IL-1β upon exposure to intestinal *SFB*. This IL-1β, in conjunction with IL-6, TGF-β, and SAA, promotes *SFB*-specific Th17 cell differentiation [39]. Additionally, Sano et al. observed increased expression of surface markers associated with DC maturation—such as CD80, CD86, MHCII, and OX40L—following exposure of bone marrow-derived dendritic cells (BMDCs) to ApoSAA. They suggested that SAA may stimulate dendritic cells to produce IL-23, thereby maintaining Th17 activation and survival [43]. Furthermore, Th17-derived IL-17 can limit SFB expansion by inducing the production of antimicrobial peptides (AMPs) and ROS [44,45]. The aberrant activation of Th17 cells and the resulting pro-inflammatory environment have been implicated in the immunopathogenesis of RSA. Given the pivotal role of SFB in promoting Th17 differentiation and sustaining IL-17-mediated immune responses, it is plausible that abnormal SFB colonization or activity may contribute to excessive Th17 activation in susceptible individuals, thereby disrupting maternal immune tolerance during early pregnancy. Although direct evidence linking SFB to RSA is currently limited, the mechanistic insights into SFB-mediated Th17 induction provide a potential avenue for exploring microbial triggers of immune imbalance in RSA pathophysiology. Future studies investigating the presence and activity of SFB in RSA patients may help clarify their role in pregnancy outcomes.

*Bacteroides fragilis* (*B. fragilis*) oppositely plays a role in modulating immune responses by inhibiting the differentiation of Th17 cells and promoting the differentiation of Treg cells through the activation of the TLR pathway in T lymphocytes. TLRs, which are pattern-recognition receptors (PRRs), identify various microbial components to eliminate pathogens, with TLR1, TLR2, and NOD2 being primarily responsible for recognizing *B. fragilis* [46]. When human peripheral blood mononuclear cells (PBMCs) are stimulated by heat-killed *B. fragilis*, they produce high levels of IL-6 and IL-8, moderate levels of IL-1β and TNF-α, and low levels of IL-10, IL-17, IL-23, and IFN-γ [46]. The immunomodulatory effects of *B. fragilis* are largely attributed to polysaccharide A (PSA), a molecule in its peritrophic capsule that aids in establishing host–microbe symbiosis and maintaining immune homeostasis [47]. Studies have shown that PSA inhibits Th17 cell differentiation primarily through TLR signaling intrinsic to CD4^+^ T cells [48]. Additionally, PSA suppresses the production of Th17-inducible cytokines and is crucial for the differentiation of CD4^+^ T cells into IL-10-producing Foxp3^+^ Treg cells. The effect of PSA on Treg differentiation depends on the presence of TLR2 on CD4^+^ T cells; TLR2-deficient T cells are unable to produce IL-10 in response to PSA stimulation [49]. The gut microbiota of patients with intrahepatic cholestasis of pregnancy (ICP) is predominantly characterized by an abundance of *B*. *fragilis* [50], highlighting the potential impact of this species on pregnancy outcomes. Similarly, considering that RSA is also closely linked to immune dysregulation—particularly Th17/Treg imbalance—*B. fragilis* may exert a protective effect by restoring immune homeostasis. Its capacity to inhibit pro-inflammatory Th17 responses while enhancing regulatory T cell pathways points to a potential immunoregulatory role in preventing RSA. However, further studies are needed to confirm the presence and function of *B. fragilis* in RSA patients and to explore its therapeutic potential.

*Bifidobacterium bifidum* has been shown to significantly influence the balance between Th1 and Th2 responses, Th17 cell polarization, and the activation of CD8^+^ T cell effectors in vivo. *Bifidobacterium species* include *bifidobacterium longum*, *bifidobacterium shortum*, *bifidobacterium bifidum*, and *bifidobacterium animalis*. These bacteria are early colonizers of the gut and play a crucial role in the maturation of the human immune system through direct interactions with immune cells and regulation of innate and adaptive immune pathways. *Bifidobacterium* strains were effective in fully maturing DCs, although the levels of cytokine production varied. Some upregulate IL-10, TNF-α, IFN-γ, and some upregulate IL-17. Different species play different roles in Th1/Th2 and Th17/Treg balance [51]. Overall, Bifidobacteria are involved in both inflammatory responses and host immunomodulation. Importantly, dysregulation of these immune pathways has been closely associated with the pathogenesis of RSA. Therefore, the immunomodulatory functions of *Bifidobacterium*, especially their ability to restore Th1/Th2 and Th17/Treg balance, suggest a potential protective role against RSA and highlight them as promising targets for therapeutic intervention in affected individuals [52]. Tersigni et al. demonstrated that oral administration of *Bifidobacterium longum* ES1 could reduce gut permeability, lower serum LPS levels, and concurrently alleviate endometrial inflammation in women with RSA [53]. Moreover, beyond RSA patients, maternal supplementation with *Bifidobacterium bifidum* has been shown to influence immune system development and intestinal tissue maturation in offspring, further underscoring the long-term immunological impact of these beneficial microbes [54,55].

There is evidence suggesting that gut microbiota-derived metabolites play a crucial role in regulating Th17 and Treg cell homeostasis. Notably, adenosine triphosphate (ATP) and SCFAs have been extensively studied. ATP from gut microbiota activates myeloid and lymphoid cells via the P2X receptor and lamina propria cells (CD70^high^CD11c^low^) via P2X and P2Y receptors. This activation promotes the expression of pro-inflammatory cytokines, enhances Th17 cell development, and inhibits Treg cell development [51]. In contrast, SCFAs, produced by anaerobic intestinal bacteria fermenting dietary fibers, regulate the development of intestinal Treg cells. SCFAs bind to GPR43 and activate NLRP3 inflammasomes, promoting cell maturation and the secretion of IL-1β and IL-18 [51].

ATP has been shown to regulate immune cell function through ATP sensors, including P2X and P2Y receptors. Atarashi et al. demonstrated that ATP derived from gut microbiota activates a specific subpopulation of CD70^high^CD11c^low^ lamina propria cells via P2X and P2Y receptors. These cells express various molecules, such as IL-6, IL-23p19, and TGF-β, which promote Th17 cell differentiation and activate integrins αV and β8 [56]. There is additional evidence that the P2X7R signaling pathway in visceral adipose tissue (VAT) also contributes to the TH17-polarized environment. After stimulation with P2X7R, there was a significant increase in IL-1β, IL-6, and IL-17 in lean donor explants [57]. Activation of P2X7 receptors by extracellular ATP leads to the assembly of the NLRP3 inflammasome and the release of active IL-1β and IL-18 in a caspase-1-dependent manner. This process ultimately inhibits IL-10 production by Th17 cells, affecting their differentiation and memory [58,59]. However, it remains unclear if ATP from gut microbiota has a similar role. Further research is needed to elucidate the mechanisms underlying the development and functional maturation of gut microbiota and Th17 cells.

Binding of gut microbial-derived SCFAs to non-hematopoietic FFAR2 receptors appears to stimulate potassium (K^+^) efflux and hyperpolarization, leading to the activation of NLRP3 inflammasomes and alterations in the local cytokine environment [60,61]. This modulation affects the size and function of the colonic Treg cell pool. However, direct evidence remains limited and requires further investigation [51]. Clostridial species from groups IV and XIVa have been shown to promote the induction of Tregs in the cecum and proximal colon, contributing to their accumulation in the colonic lamina propria [62]. This effect may be linked to SCFAs production by these clostridial microbiota. SCFAs can induce the production of TGF-β1 by epithelial cells, promoting the initial induction of peripheral Tregs [63]. SCFAs can induce the production of TGF-β1 by epithelial cells, promoting the initial induction of peripheral Tregs. Butyrate, a key SCFA, induces thymic Treg cell proliferation through GPR43, a member of the G protein-coupled receptor family [64]. Additionally, butyrate inhibits histone deacetylase (HDAC) and promotes acetylation of histone H3 at the Foxp3 enhancer, facilitating the differentiation of CD4^+^ T cells into peripheral Tregs [64]. Besides its effects on Tregs, butyrate also inhibits dendritic cell activation by suppressing RelB expression in the NF-κB signaling pathway [65] and induces the expression of several anti-inflammatory genes in GPR109a-dependent dendritic cells [66].

The gastrointestinal tract (GI) contains most of the body’s serotonin (5-hydroxytryptamine, 5-HT), but the mechanisms about gut-derived 5-HT metabolism are unclear. 5-HT is not a direct metabolite of the gut microbiota, but its biosynthesis is largely regulated by it. Indigenous spore-forming bacteria (Sp) from mouse and human microbiota promote serotonin biosynthesis in colonic enterochromaffin cells (ECs), which provide serotonin to mucous membranes, lumens, and circulating platelets [67]. Gut microbes regulate 5-HT levels by inducing tryptophan hydroxylase 1(TPH1) expression through SCFAs [68]. Moreover, it was found that microbiome-mediated host 5-HT regulated gastrointestinal peristalsis and platelet function [67]. Intestinal 5-HT stimulates the production of cytokines from DC and regulates the ability of DC to activate CD4 T cells [69]. It can also enhance IL-10 production through different regulatory CD4^+^ T cell subsets, thereby improving Treg function [70].

#### 4.1.3. Macrophages Polarization and Trophocyte Invasion

Fetal trophoblast and maternal placental immune cells interact at the mother–fetal interface. Macrophages are essential decidual immune cells for normal pregnancy. Trophoblast and macrophage work together to maintain the normal pregnancy process, and the two regulate each other’s functional roles. RSA may be associated with trophoblast dysfunction, and macrophage polarization and its metabolic abnormalities. Macrophages are biased towards the M1 phenotype in spontaneous abortion. It is possible that Fas/FasL mediating apoptosis of macrophages, downregulation of PPARγ expression, dysregulation of the IL-33/ST2 signaling pathway, abnormal expression of RANKL, or Akt/STAT6-Jmjd3/IRF4 signaling pathway predisposing macrophages to M1 polarization may be involved in the occurrence of abortion [71]. Recently, researchers found that Jupiter microtuleassociated homolog 2 (JPT2) expression was reduced in villus specimens from RSA patients and placentas from aborted mice. This decrease inhibits JNK phosphorylation, and then downregulates the expression of ACKR3 protein and IL-6, promoting M1 polarization and ROS accumulation in macrophages, which is not conducive to pregnancy. In addition, the lack of JPT2 also changed the metabolism of macrophages by inhibiting the secretion of IL-6, enhanced the production of citric acid, and then also promoted the M1 polarization and ROS accumulation of macrophages. The changes in macrophages further inhibit the function of trophoblastic cells and promote their apoptosis [72].

The regulation effect of gut microbiota on the immune system also occurs in the polarization of macrophages. For example, researchers have shown that increasing the gut microbiota of Lactobacillus johnsonii can help maintain intestinal integrity and promote the anti-inflammatory activity of macrophages [73]. However, articles directly demonstrating the involvement of gut microbiota in RSA through macrophage polarization and trophoblast invasion are scarce.

#### 4.1.4. Antigen Presenting Cells (APCs) System Response

APCs, including DCs, macrophages (MΦs), and monocytes, play a crucial role in pregnancy outcomes [74,75,76] and interact significantly with the gastrointestinal microbiota. Research shows that APCs are notably influenced by pathogen-associated molecular patterns (PAMPs) and damage-associated molecular patterns (DAMPs) from both regulatory and intestinal microbiota, particularly during allogeneic stem cell transplantation [77]. These cells are essential for initiating specific immune responses and inducing immune tolerance. APCs process antigens and present them on their surface in conjunction with major histocompatibility complex (MHC) class I and class II molecules during their maturation. Mature APCs also express adhesion molecules and stimuli that aid in the differentiation of early T cells. Additionally, APCs produce cytokines and chemokines necessary for T cell replication, differentiation, and activation. For example, IL-12 promotes the differentiation of T cells towards the Th1 phenotype.

Microbial components are recognized by PRRs, such as those on APCs and TLRs on epithelial cells, which then regulate the immune system in the gut. Upon the binding of TLRs to PAMPs and the insertion of antigens, APCs mature and express stimulatory molecules such as CD80, CD86, and IL-12, which are associated with T cell activation. Mature APCs present microbial antigens in association with MHC class II molecules to immature T cells, thereby initiating the acquired immune response. The innate immune response relies on MyD88, as MyD88-deficient DCs show impaired production of IFN-γ. Mature APCs then migrate to the mesenteric lymph nodes (MLNs), where they use cytokine patterns to drive the differentiation of CD4^+^ Th0 cells into various Th cell subtypes, as mentioned above [78]. Additionally, cytotoxic T lymphocytes are activated to enhance the cellular immune response and promote phagocytosis by Th1 cells. Cytokines and T cells subsequently enter the bloodstream and migrate to the liver and spleen, where they further regulate immune system functions [27].

#### 4.1.5. Autoimmune

Elevated plasma levels of antiphospholipid antibodies are commonly observed in patients with autoimmune RSA. There is increasing evidence suggesting that cross-reactivity to microbial antigens might trigger such autoimmune responses. For instance, Ruff et al. recently found that the DNA methyltransferase from *Roseburia* intestinalis, a predominant commensal species, shares homology with human β2-glycoprotein I. This molecular similarity could potentially drive the production of autoreactive Th1 cells and autoantibodies in patients with antiphospholipid syndrome [79].

Antiphospholipid antibodies encompass various autoantibodies directed against phospholipids, including anticardiolipin (ACA), anti-β2-glycoprotein I (GP), antiphosphatidylglycerol, lupus anticoagulant, antiphosphatidylserine, antiphosphatidylinositol, and antiphosphatidic acid [80]. Additionally, antibodies against thyroid antigens, antinuclear antibodies (ANAs), antithrombinogen antibodies, and anti-laminin antibodies have also been linked to pregnancy complications [81,82].

During pregnancy, hormonal changes can lead to a hypercoagulable state, affecting blood rheology and increasing the risk of thrombotic events. This heightened coagulability can cause microthrombosis in uterine spiral arteries or chorionic vessels, resulting in multiple placental infarctions. These infarctions impair uterine–placental blood flow, leading to placental ischemia and hypoxia, which can adversely affect both maternal health and pregnancy outcomes.

An imbalance between tissue plasminogen activator (t-PA) and plasminogen activator inhibitor 1 (PAI-1) further exacerbates the risk of thromboembolism and fetal hypoxia, contributing significantly to RSA. Elevated t-PA/PAI-1 ratios or increased PAI-1 activity indicate a pre-thrombotic state and elevate the risk of miscarriage. In addition, Yao et al. [82] found that RSA was related to the decrease of activated partial prothrombin time (APTT) and prothrombin time (PT), and the increase of fibrinogen (FIB) and D-dimer (DD). Thromboelastogram (TEG) shows that elevated coagulation angle (α) and maximum thrombotic amplitude (MA) values indicate an increased risk of multiple miscarriage.

Notably, a negative correlation has been observed between the abundance of Odoribacter in the intestinal microbiota and PAI-1 levels. Odoribacter can reduce PAI-1 synthesis by fermenting carbohydrates to produce butyrate, which alleviates vasoconstriction and endothelial dysfunction [83]. However, the specific differences in Odoribacter levels between RSA patients and healthy controls require further clinical investigation through fecal sample analysis.

Metabolites from gut microbiota were proved to play a role in thrombosis and cardiovascular events. Dietary choline sources, including phosphatidylcholine, choline, betaine, and carnitine, are metabolized by gut microorganisms into trimethylamine (TMA). TMA is then oxidized by the hepatic enzyme flavin monooxygenase 3 (FMO3) to form TMAO [84]. Elevated plasma levels of TMAO are positively correlated with the development of atherosclerosis and influence platelet function. TMAO interacts with platelets, altering calcium signaling and increasing platelet hyperreactivity, thereby raising the risk of thrombosis [85,86]. Research has shown that TMAO can induce the expression of tissue factor and other coagulation factors in endothelial cells, enhancing their procoagulant activity [87]. It also activates NF-κB nuclear factor in vivo and in vitro, leading to increased vascular perfusion and macrophage adhesion. Additionally, TMAO reduces the expression of Cyp7a1, a key enzyme in bile acid synthesis, and impairs cholesterol transport, resulting in cholesterol accumulation, foam cell formation, and platelet hyperreactivity [88].

Furthermore, gut microbiota-derived endocrine-like products, which can act on distant organs via the bloodstream, modulating endocrine functions, can influence the body’s endocrine system, indirectly affecting hormones such as cortisol and leptin, which are associated with URSA [1].

### 4.2. Damage to the Maternal–Fetal Interface

LPS injections, a structural component of gut microbiota, are frequently used in experimental models to study pregnancy complications such as fetal growth restriction, thrombosis, preterm labor, and miscarriage. Inflammation induced by LPS has been identified as a critical factor in miscarriage associated with placental tissue damage. Luna et al. observed that 2 h after LPS exposure, histopathological analysis of the placenta revealed significant injury to the critical maternal–fetal interface in the placental labyrinth zone [89]. Endothelial cells in this region exhibited edema and hemorrhage, which could lead to placental bleeding events. Additionally, low levels of key proteins like the adhesion molecule P-Sel and placental growth factor in the placental tissue may contribute to pregnancy complications [90]. Endothelial dysfunction has been linked to increased P-Sel expression, which is crucial for physiological processes during pregnancy. Studies have shown that LPS injury causes detachment of endothelial cells from the basal lamina and damage to spongy trophoblast cells, resulting in impaired giant trophoblast cells. Notably, P-Sel expression in the spongy trophoblast region of the placental labyrinth is significantly reduced in LPS-stimulated models [89], potentially contributing to URSA.

## 5. Potential Treatment Strategies

The findings discussed above offer valuable insights into the pathogenesis of URSA and suggest new directions for clinical interventions and treatment strategies.

### 5.1. Treatment with Probiotics

Probiotics may play a significant role in regulating gut microbiota and potentially influencing pregnancy outcomes. The use of various probiotic strains has been associated with the development or stimulation of Th2-mediated immune responses, which can exacerbate or inhibit atopic diseases. Some probiotics have been shown to stimulate Th2 responses, which can inhibit Th2-mediated atopic diseases, offering potential therapeutic benefits for conditions like atopic dermatitis and allergic rhinitis [27]. However, Alipour R. et al. found that treatment with vaginal Lactobacillus casei probiotics did not fully restore the dysregulated expression of TLR2 and TLR4, suggesting that other Lactobacillus strains may need to be explored [91]. Supporting this, a recent study by Martínez-Villaluenga et al. demonstrated that administration of *Ligilactobacillus salivarius* CECT 30632 improved pregnancy rates among women with infertility (55%) and RSA (80%). This highlights the importance of strain specificity and local microbiota modulation in improving reproductive outcomes [92]. Probiotics can also suppress inflammation by inhibiting various signaling pathways, such as the NF-κB pathway. This inhibition may be linked to changes in the mitogen-activated protein kinase (MAPK) and PRRs pathways. Certain probiotics can control checkpoints in the MAPK pathway, suggesting that both NF-κB and MAPKs play roles in pro-inflammatory cytokine production [27]. Rafiee M. et al. found that probiotic treatment can ameliorate disturbed HLA expression, as well as abnormal APCA and IgG production, in couples with RSA [93]. Thus, the use of probiotics may target these pathways, providing anti-inflammatory effects. Additionally, the modulation of gastrointestinal microbial compounds by probiotics may influence mucosal and systemic immunity [94]. This regulation could be crucial in addressing the underlying immune dysfunctions associated with URSA, potentially leading to improved pregnancy outcomes. Further research into the specific strains and mechanisms of probiotics could pave the way for targeted treatments in managing RSA.

### 5.2. High-Fibre Diet Therapy

Dietary fiber can be metabolized by intestinal microbiota to produce SCFAs [95], which, as mentioned earlier, is beneficial for maintaining a normal pregnancy. Dietary interventions have been shown to improve fungal composition by reducing the fungal microbiota associated with GDM [14]. Therefore, consider using a high-fiber diet to improve RSA. At present, relevant research is lacking, and further exploration is needed.

### 5.3. Fecal Microbial Transplantation Treatment

In addition to probiotic administration, microbiota fecal transplantation (FMT) has emerged as a significant research area. FMT has demonstrated effectiveness in treating various conditions, including ulcerative colitis, Crohn’s disease, steroid-resistant acute graft-versus-host disease, hepatic encephalopathy, functional gastrointestinal disorders, and certain blood disorders [51]. Although FMT has not been extensively studied in patients with RSA, there is a reported case in which a patient received a vaginal microbiota transplant (VMT) and subsequently delivered a healthy baby [96]. This highlights the possible role of microbiota transplantation as an emerging therapeutic strategy for RSA. Despite its promise, FMT faces several challenges before it can be widely adopted in clinical settings. Several safety concerns must be addressed before it can be considered for clinical application in this population. These include potential risks of pathogen transmission, immune rejection, and unintended microbiome shifts, as well as a lack of standardized donor screening and preparation protocols. A deeper understanding of the relationship between specific gut microbiota and host health is crucial for advancing FMT therapies. This knowledge will help in developing targeted FMT treatments that could potentially be applied for managing RSA by restoring a healthy gut microbiome and improving pregnancy outcomes. Future research should focus on clarifying the causal relationship between gut dysbiosis and RSA, identifying microbial biomarkers predictive of miscarriage risk, and conducting well-designed clinical trials to assess the efficacy and safety of FMT specifically in RSA patients [56].

### 5.4. Lipid Emulsion Treatments

The link between gut microbiota and pregnancy outcomes is largely mediated by the immune system, suggesting that treatments could target this connection. One such treatment involves intralipid (fat emulsion), commonly used for parenteral nutrition. The active ingredient in intralipid has been found to decrease Th1 cytokine and NK cell activity while promoting trophectodermal cell invasiveness. A prospective, randomized, controlled trial demonstrated that both intralipid injections and intravenous immunoglobulin (IVIG) decreased NK cell concentrations and improved pregnancy success rates. The comparison between the two treatments showed no statistically significant difference, indicating that intralipid injections could be a viable alternative to IVIG, which is expensive and associated with numerous adverse effects [97]. In conclusion, while intralipid shows promise as an alternative treatment for URSA, more data are needed to support its effectiveness in increasing successful pregnancy rates. Further studies on sustained pregnancies and live births are essential to validate these preliminary findings and establish reliable treatment protocols.

### 5.5. Traditional Chinese Medicine (TCM) Treatments

TCM offers a unique approach to treating RSA, particularly in cases characterized by kidney deficiency and blood stasis. The basic treatment principle involves replenishing essence and marrow, as well as invigorating blood. Studies have indicated that herbs used to strengthen the spleen and stomach can enhance the structure and abundance of the intestinal microbiome, thereby promoting homeostasis. After entering the intestinal tract, the half-life and bioactivity of Chinese herbal ingredients are influenced by the bacterial microbiota, often acting as prebiotics to improve the abundance of beneficial bacteria, which aids in drug absorption and efficacy. One TCM formula used for this purpose is a modified version of Shou Zi Wan, which tonifies the kidney and tranquilizes the fetus. This formula, composed of Astragalus, Cuscuta, Sambucus, Radix et Rhizoma Gastrodiae, Fructus Schisandrae, Salviae Miltiorrhizae, and Colla Corii Asini, has been shown to positively affect the Th1/Th2 cytokine balance in patients with early onset of premenstrual miscarriage due to renal deficiency [1]. Xu Guangli et al. observed that using a formula to tonify the kidneys and invigorate blood led to a decrease in enterococci, Enterobacteriaceae, and yeasts, while increasing the number of Bifidobacteria and Lactobacillus. This change was accompanied by an improvement in the TCM symptom scores in the intestines of RSA patients [98]. Additionally, another study reported that the Shoutai pill combined with peony and licorice decoction (including dodder, mulberry parasitism, intermittent, codonopsis, yam, white art, white peony, etc.) effectively improved the term delivery rate in patients with spleen and kidney qi deficiency syndrome [19]. These findings suggest that TCM treatments, by modulating the intestinal microbiome and restoring immune balance, may offer a promising complementary approach to managing RSA. However, it is important to acknowledge that high-quality clinical evidence supporting the efficacy of TCM for RSA remains limited. Variability in herbal composition, dosing, and patient syndrome classification poses challenges in standardizing and validating treatments. Moreover, the safety of long-term herbal interventions in pregnant women needs further exploration, particularly regarding herb–drug interactions and fetal impact. Further research and clinical trials are needed to fully understand the mechanisms and optimize the use of these herbal formulas in preventing recurrent pregnancy loss. Large-scale, randomized controlled trials are needed to systematically evaluate the efficacy, mechanisms, and safety of TCM interventions in the context of RSA.

In summary, although novel strategies offer exciting possibilities for modulating the gut microbiota and improving immune balance in RSA patients, current evidence remains preliminary. Clinical application of these therapies must proceed with caution, given the limited data on long-term safety, efficacy, and underlying mechanisms. Longitudinal studies can help determine the sequential relationship between changes in gut flora and pregnancy outcomes. One perspective is that gut dysbiosis may trigger immune dysfunction, while another suggests that immune dysfunction may lead to gut dysbiosis. Additionally, molecular biology and cell biology studies can elucidate mechanisms, including how the gut microbiota regulates the immune response and how the immune system affects the gut microbiota.

There are various treatment options available for RSA of different etiologies, though the effectiveness of many medical interventions remains controversial. Current treatments target hypothesized risk factors for miscarriage, and interventions should be based on the benefit–risk ratio of the proposed treatment. Continued research will provide valuable information for developing future interventions and treatment strategies, ultimately aiming to improve pregnancy outcomes in patients with URSA.

## 6. Conclusions

In summary, this review provides an overview of the current understanding of gut microbiota composition and function, the definition and mechanisms of recurrent spontaneous abortion (RSA), and the emerging evidence on the differences in gut microbiota and metabolites between RSA patients and healthy pregnancies. Increasing evidence suggests that gut dysbiosis and its metabolic disturbances may contribute to the pathogenesis of RSA through immune dysregulation, inflammation, and altered endocrine signaling. Despite these insights, the exact mechanisms remain incompletely understood, and most existing data are based on preclinical or observational studies.

Potential therapeutic strategies such as probiotics, fecal microbiota transplantation (FMT), and herbal medicines are promising but require further validation in well-designed clinical trials. Moreover, future studies should aim to delineate the causal relationships between gut microbiota alterations and RSA, identify specific microbial or metabolic biomarkers, and develop personalized microbiota-targeted interventions.

Understanding the gut microbiota’s role in RSA may open new avenues for diagnosis, prevention, and treatment, ultimately improving reproductive outcomes in affected women.

## Figures and Tables

**Table 1:** Research about relationship between gut microbiota, metabolites, and RSA.

**Gut Microbiota**								
**Author**	**Year**	**Group**	**Nation**	**Specimen Type**	**Composition: Phylum/Genus/Species**	**Mechanism**	**Conclusion**	**Reference**
Xia Meiyan et al.	2021	Abortion group: RSA patients (*n* = 63); control group: healthy abortive pregnant women (*n* = 60); normal group: patients who underwent intra-pelvic surgery and had no endometriosis lesions (*n* = 53)	China	faeces	Content of yeast, *Enterococci*, and *Enterobacteri*: abortion group > control group > normal group; content of *Lactobacillus* and *Bifidobacterium*: normal group > control group > abortion group	/	All patients with RSA had high levels of gut microbiota; yeast, *Enterococci*, and *Enterobacteria* were positively correlated with RSA (*p* < 0.05); *Lactobacillus* and *Bifidobacterium* were negatively correlated with RSA (*p* < 0.05)	[18]
Yongjie Liu et al.	2021	Case group: RSA patients (*n* = 41); control group: normal early-pregnant women who chose abortion (*n* = 19)	China	faeces	The abortion group was rich in firmicutes and the control group was rich in *Proteobacteria*. In the control group, *Prevotella*, *Prevotella _1*, and *Proteobacteria C* were the most abundant microflora.Abortion group Spirochetes, Fibromyces, Softenicutes ↑*; *Prevotella family NK3B31_ group*, *Bacteroideformes S24_7_Group*, *Eubacterium ruminant group*, etc. ↑*; *Prevotella (_1)*, *Prevotella (UCG_003)*, *Rothella (_1)*, and *Selenomonas (_1)* are among 48 species of bacteria ↓*	Bacterial abundance was significantly correlated with the changes in inflammatory factors and metabolites, such as IL-2, IFN-γ, IL-17A, 7-hydroxy-3-oxycholic acid, 1, 4-methylimidazolacetic acid, imidazolpropionic acid, etc	The abundance and uniformity of intestinal bacteria in abortion patients were low, and the gut microflora was obviously clustered. The proportion of some bacteria increased; *Prevotella_1*, *Prevotella_UCG_003* and *Selenomonas_1*, which are the dominant bacterial groups in the gastrointestinal environment of healthy people, were significantly reduced	[21]
Zhi Li et al.	2024	Case group: RSA patients (*n* = 12); control group: normal early-pregnant women who chose abortion (*n* = 15)	China	faeces	The top 5 dominant genera in the case group were: *Clostridium_sensu_stricto_1_unclassified*, *Escherichia—Shigella_unclassified*, *Klebsiella_pneumoniae*, *Streptococcus_salivarius*, *uncultured_Klebsiella_sp.*, *Salivarius*, *uncultured _Klebsiella_sp.* at the species level. The top 5 dominant genera in the control group were, in order, *Megamonas_unclassified*, *Bacteroides _unclassified*, *Agathobacter_unclassified*, *Faecalibacterium_unclassified*, and *Bacteroides_uniformis*	/	Gut microbiota of URSA patients has decreased diversity and changes in the dominant species.	[3]
Ying Cui et al.	2021	NR group: pregnant women who terminated their pregnancy and did not have a history of spontaneous abortion (*n* = 30) RSA group: RSA patients (*n* = 30)	China	faeces	The bacterial abundance index decreased in RSA patients, but the bacterial diversity index increased. They also found that *Roseburia* significantly decreased while *Ruminococcus* significantly increased in RSA patients. Also, in RSA patients with intrauterine adhesion, PCOS, and BMI > 23.9, *Klebsiella* significantly increased, and *Prevotella.9* and *Roseburia* significantly decreased.	Functional prediction analysis indicated that gut microbiota may play their role through membrane transport, carbohydrate metabolism, amino acid metabolism, and other mechanisms.	RSA patients have abnormal gut microbiota compared with normal pregnant women. Butyrate-producing bacteria, like *Roseburia, Prevotella.9*, and *Agathobacter*, may play an important role in pregnant women, and are associated with RSA.	[22]
**Metabolite**								
**Author**	**Year**	**Group**	**Nation**	**Specimen type**	**Constitution**	**Mechanism**	**Conclusion**	**Reference**
Yongjie Liu et al.	2021	Case group: RSA patients (*n* = 41); control group: normal early-pregnant women who chose abortion (*n* = 19)	China	faeces	239 differentiated metabolites were found in the miscarriage group compared to the control group. In miscarried patients, bile acids, methyl dihydrophosphonate, 3a, 7a, 12b-trihydroxy5b-cholic acid, 3a, 6a, 7b-trihydroxy5b-cholic acid, 3a, 6a, 7b-trihydroxy5b-cholic acid, 3α -hydroxy-5-β-chol-8,14-diene-24-cholic acid, 3,8-dihydroxy-6-methoxy-7(11)-dibenzyloxyphenol-12,8-lactone, d -urobilinogen, 1b,3a,7b-trihydroxy5b-cholic acid, THA and goose deoxycholic acid sulfate. ↑*	Metabolites such as 1,4-methylimidazole acetic acid in stool were positively correlated with IL-17A, IL-17F, TNF-α and IFN-γ. ROC analysis showed that imidazollic acid and 1,4-methylimidazolacetic acid were significantly associated with abortion	There were four broad clusters of differential metabolites: (1) glycerophospholipids and aryl alcohol lipids: control group > abortive group; (2) steroids and their derivatives; (3) amino acids and their derivatives; (4) alkaloids, drugs, and other metabolites: abortive group > control group. Differential metabolites were associated with the following metabolic processes: (1) bile secretion; (2) histidine metabolism; (3) glycerophospholipid metabolism; (4) arachidonic acid metabolic pathway; (5) steroid hormone biosynthesis.	[21]
Zhi Li et al.	2024	observation group: URSA patients (*n* = 12); control group: normal early-pregnant women who chose abortion (*n* = 15)	China	faeces	Acetic acid, propionic acid, butyric acid, deoxycholic acid (DCA) and glycolic acid (GLCA) ↓* in observation group.	DCA, GLCA, acetate, propionate, and butyrate were positively correlated with Tregs and Bregs frequencies. GLCA and butyrate were negatively correlated with Th1 and Th17 frequencies. Propionate and butyrate were negatively correlated with plasma B cell frequency	Levels of DCA, GLCA, acetate, propionate, and butyrate of intestinal microbial origin were decreased in URSA.	[3]

Table 1 lists the results of all published clinical research about gut microbiota and gut microbiota or metabolites up to September 2024. ↑ indicates increase; ↓ indicates decrease; * indicates statistically significant difference (*p* < 0.05).

## Data Availability

The original data presented in the study are openly available in PubMed and CNKI.

## Abbreviations

The following abbreviations are used in this manuscript:

RSA	Recurrent Spontaneous Abortion
IL	Interleukin
IFN-γ	Interferon Gamma
TNF-α	Tumor Necrosis Factor Alpha
SCFA	Short-Chain Fatty Acids
TCM	Traditional Chinese Medicine
NK	Natural Killer Cells
Th	T Helper Cells
Treg	Regulatory T Cells
Breg	Regulatory B Cells
DC	Dendritic Cells
APC	Antigen-Presenting Cells
LPS	Lipopolysaccharide
PRR	Pattern Recognition Receptor
MAPK	Mitogen-Activated Protein Kinase
NF-κB	Nuclear Factor Kappa B
TLR	Toll-Like Receptor
SOCS	Suppressor of Cytokine Signaling
ATP	Adenosine Triphosphate
NLRP3	NOD-, LRR- and Pyrin Domain-Containing Protein 3
FMT	Fecal Microbial Transplantation
TMA	Trimethylamine
TMAO	Trimethylamine N-Oxide
FMO3	Flavin Monooxygenase 3
HDAC	Histone Deacetylase
BCAA	Branched-Chain Amino Acids
DD	D-Dimer
APTT	Activated Partial Prothrombin Time
PT	Prothrombin Time
FIB	Fibrinogen
TEG	Thromboelastogram
MA	Maximum Thrombotic Amplitude
PAI-1	Plasminogen Activator Inhibitor 1
T-PA	Tissue Plasminogen Activator
DCA	Deoxycholic Acid
FFAR2	Free Fatty Acid Receptor 2
GPCR	G Protein-Coupled Receptors
TPH1	Tryptophan Hydroxylase 1
MDPI	Multidisciplinary Digital Publishing Institute
DOAJ	Directory of Open Access Journals
TLA	Three Letter Acronym
LD	Linear Dichroism
URSA	Unexplained Recurrent Spontaneous Abortion
